# Supplementary material for: Searching for the cellular underpinnings of the selective vulnerability to tauopathic insults in Alzheimer’s disease
Source: Commun Biol. 2025 Feb 7;8:195. doi: 10.1038/s42003-025-07575-1 (PMC11806020; doi:10.1038/s42003-025-07575-1)
Supplement: Supplementary file 4 — Description of Additional Supplementary File [file 42003_2025_7575_MOESM4_ESM.pdf]

Description of Additional Supplementary Files

File name: Supplementary Data 1

Description: The source data behind the graphs in the paper

File name: Supplementary Data 2

Description: Gene lists for SV-G (S10), SV-C (S11), SR-G (S12), and SR-C (S13) gene ontology (GO) analysis (Figure 7; Figure S21), as well as for the differentially expressed genes in the cortical (S14) and hippocampal (S15) glutamatergic neurons (Figure S22).
